# Phenotypic and molecular analyses in diploid and tetraploid genotypes of *Solanum tuberosum* L. reveal promising genotypes and candidate genes associated with phenolic compounds, ascorbic acid contents, and antioxidant activity

**DOI:** 10.3389/fpls.2022.1007104

**Published:** 2023-01-18

**Authors:** Jhon A. Berdugo-Cely, María del Socorro Céron-Lasso, Roxana Yockteng

**Affiliations:** ^1^ Corporación Colombiana de Investigación Agropecuaria-AGROSAVIA, Centro de Investigación Turipaná, Km 13 vía Montería-Cereté, Montería, Córdoba, Colombia; ^2^ Corporación Colombiana de Investigación Agropecuaria (AGROSAVIA), Centro de Investigación Tibaitatá, Km 13 vía Mosquera-Bogotá, Mosquera, Cundinamarca, Colombia; ^3^ Institut de Systématique, Evolution, Biodiversité-UMR-CNRS 7205, National Museum of Natural History, Paris, France

**Keywords:** antioxidants, phenolic compounds, ascorbic acid content, antioxidant activity, GWAS, potato, diploid, tetraploids

## Abstract

Potato tubers contain biochemical compounds with antioxidant properties that benefit human health. However, the genomic basis of the production of antioxidant compounds in potatoes has largely remained unexplored. Therefore, we report the first genome-wide association study (GWAS) based on 4488 single nucleotide polymorphism (SNP) markers and the phenotypic evaluation of Total Phenols Content (TPC), Ascorbic Acid Content (AAC), and Antioxidant Activity (AA) traits in 404 diverse potato genotypes (84 diploids and 320 tetraploids) conserved at the Colombian germplasm bank that administers AGROSAVIA. The concentration of antioxidant compounds correlated to the skin tuber color and ploidy level. Especially, purple-blackish tetraploid tubers had the highest TPC (2062.41 ± 547.37 mg GAE), while diploid pink-red tubers presented the highest AA (DDPH: 14967.1 ± 4687.79 μmol TE; FRAP: 2208.63 ± 797.35 mg AAE) and AAC (4.52 mg ± 0.68 AA). The index selection allowed us to choose 20 promising genotypes with the highest values for the antioxidant compounds. Genome Association mapping identified 58 SNP-Trait Associations (STAs) with single-locus models and 28 Quantitative Trait Nucleotide (QTNs) with multi-locus models associated with the evaluated traits. Among models, eight STAs/QTNs related to TPC, AAC, and AA were detected in common, flanking seven candidate genes, from which four were pleiotropic. The combination in one population of diploid and tetraploid genotypes enabled the identification of more genetic associations. However, the GWAS analysis implemented independently in populations detected some regions in common between diploids and tetraploids not detected in the mixed population. Candidate genes have molecular functions involved in phenolic compounds, ascorbic acid biosynthesis, and antioxidant responses concerning plant abiotic stress. All candidate genes identified in this study can be used for further expression analysis validation and future implementation in marker-assisted selection pre-breeding platforms targeting fortified materials. Our study further revealed the importance of potato germplasm conserved in national genebanks, such as AGROSAVIA’s, as a valuable genetic resource to improve existing potato varieties.

## Introduction

Studies on the functional properties of phytochemicals such as polyphenols, carotenoids, and vitamins are extensive because of their beneficial effect on human health (Valcarcel et al., 2016; Fraga et al., 2019; Lourenço et al., 2019). Polyphenols and ascorbic acid (vitamin C) are known to be powerful antioxidants that may scavenge Reactive Oxygen Species (ROS) to prevent DNA damage (Murniece et al., 2014; Silva-Beltrán et al., 2017; Pawlowska et al., 2019). Plants produce polyphenols (*e.g.*, phenolic acids, stilbenes, coumarins, lignins, flavonoids, among others) as secondary metabolites with antioxidative properties, that are involved in defense against ultraviolet radiation, pathogen attack, stress responses, plant growth, development, immunity, and allelopathy. In humans, polyphenols have an effect in the cell proliferation, metabolism, weight, and chronic disease (Cory et al., 2018; Fraga et al., 2019; Lourenço et al., 2019). Ascorbic acid is an essential water-soluble micronutrient. It is one of the main redox components with properties as an enzyme cofactor in many metabolic pathways such as the biosynthesis of organic components, regulatory processes, hormone production, and signal transduction. In humans, this compound supports the immune system by acting in the activation and stabilization of the B and E vitamins and folic acid. In plants, ascorbic acid plays a role in plant development, photosynthesis regulation, cell division, and abiotic stress tolerance (Bozonet and Carr, 2019; Pawlowska et al., 2019; Liu et al., 2020).

Humans must consume polyphenols and vitamins through their diet since most cannot synthesize them (Chambial et al., 2013; Pawlowska et al., 2019). These compounds are produced by several fruits and vegetables, such as potato (*Solanum tuberosum* L.). Potatoes are the fourth most important food crop worldwide after corn, rice, and wheat, with a consumption per capita of 32.4 kg in 2019 (FAOSTAT, 2022). Potato is valuable for food security and nutrition because it contains proteins, carbohydrates, lipids, organic acids, polyphenols, glycoalkaloids, fibers, minerals, and vitamins (Singh and Kaur, 2009; Kowalczewski et al., 2019) and especially because it is by far eaten more than other vegetables (Navarre et al., 2009). A fresh red potato of 100 g without skin can contain 2.06 g of proteins, 16.4 g of carbohydrates, 13.8g of fiber, 5mg of Ca, 0.39mg of Fe and Zn, and 21.3gr of Vitamin C, among others components (USDA, 2022). Potato tubers are the third most important source of phenols after apples (*Malus domestica* Borkh.) and oranges (*Citrus×sinensis*); 50% of phenolic compounds accumulate in the potato peel and tissues nearby (Ah-Hen et al., 2012; Hesam et al., 2012; Murniece et al., 2014; Mishra et al., 2020).

The information about the genetic regulation of the polyphenol biosynthesis in potato tubers is scarce (Valiñas et al., 2015). Then, a few genes related to the production of phenolic compounds in potatoes, such as anthocyanin and flavonoids, have been identified through QTL analyses (Zhang et al., 2009), transcriptome analyses (Stushnoff et al., 2010; Liu et al., 2015; Liu et al., 2018), and Genome-Wide Association Studies (GWAS) (Parra-Galindo et al., 2019; Parra-Galindo et al., 2021). In plants, three pathways, the shikimate, phenylpropanoid, and flavonoid pathways, have a function in the biosynthesis of phenolic compounds (Patil and Masand, 2018). In particular, the genes PAL (*phenylalanine-ammonia-lyase*), C4H (*cinnamate 4-hydroxylase*), 4CL (*4-coumarate-CoA ligase*), CHS (*chalcone synthase*), HCT (*hydroxycinnamoyl-CoA shikimate*), HQT (*hydroxycinnamoyl-CoA quinate*) and transcriptions factors (TFs) as MYB and basic helixloop-helix (bHLH) play a role in the regulation of the synthesis of these compounds (Vogt, 2010; Valiñas et al., 2015; Valcarcel et al., 2016). On the other hand, the ascorbic acid is synthesized at least by four pathways, the L-Galactose (Wheeler et al., 1998; Wheeler et al., 2003), the L-Glucose (Wheeler et al., 2003; Wolucka and Van Montagu, 2003), the D-Galacturonate (Agius et al., 2003; Wheeler et al., 2003), and the Myo-Inositol pathways (Lorence et al., 2004); where genes as DHA (*ascorbate peroxidase*), GDH (*D-galacturonate reductase*), GGP (*L-galactose dehydrogenase*), GME (*L-galactono-1,4-lactone dehydrogenase*), GMP (*GDP-D-mannose3′, 5′-epimerase*) among others have a role in the synthesis of this component (Caruso et al., 2021; Zheng et al., 2022). Yet, reports about the genes associated with ascorbic acid synthesis and its antioxidant activity in potatoes are not available. Identifying molecular markers that flank gene-related to the production of phenolic compounds and ascorbic acid in potatoes will allow the use of Marker-Assisted Selection (MAS) in breeding programs. MAS could reduce the time and cost of improving biofortified materials.

The origin, diversification, and domestication of *S. tuberosum* occurred in the South American Andean highlands around the Lake Titicaca between Peru and Bolivia (Grun, 1990). The potato´s contemporary landraces are gene pools from south Chile to north Colombia (Hawkes, 1990; De Haan and Rodriguez, 2016). Colombia is one of the secondary diversity centers of potatoes (Spooner et al., 2014), considered essential for the country’s food security, in which 3.1 million tons are produced (FAOSTAT, 2022). The Corporación Colombiana de Investigación Agropecuaria (AGROSAVIA) is responsible for conserving and characterizing the Colombian potato genetic resources (Colombian Central Collection - CCC). This collection is one of the largest and most diverse for this species in South America (Berdugo-Cely et al., 2017; Berdugo-Cely et al., 2021; Nagel et al., 2022), with more than 2000 accessions that include landraces or native genotypes (materials developed as a product of selection by Colombian farmers), commercial varieties, and wild-relative species of *S. tuberosum*. In an earlier study (Berdugo-Cely et al., 2017), 809 native genotypes of the CCC collection were analyzed using morphological characters and Single Nucleotide Polymorphism (SNP). Diploid and tetraploid genotypes were separated in two main populations distinguished by ploidy level. Furthermore, this study also found high genetic diversity in shape, flesh, and skin colors in tubers of tetraploid potatoes. The flesh and skin color of potato tubers have been correlated with polyphenols and ascorbic acid content. Tubers with dark colors presented high content of these compounds and an increase in antioxidant activity (Hejtmánková et al., 2009). The high phenotypic diversity in potato tubers conserved in the CCC allows us to believe that this germplasm has a valuable genetic resource for breeding programs to generate new cultivars with high nutritional values.

In consequence, the present study aimed 1) to analyze the natural phenotypic variation of Total Phenol Content (TPC), Ascorbic Acid Content (AAC), and Antioxidant Activity (AA) in potato tubers of diploid and tetraploid genotypes of the CCC to select promising genotypes to be used in future breeding programs, and 2) to associate this phenotypic information with SNP through GWAS to identify market-trait association which may collocate candidate genes with possible implication in the production of polyphenols and ascorbic acid and their antioxidant activity in potato. We hypothesize that the ploidy level affects the concentration and the genetic regulation of polyphenols, ascorbic acid biosynthesis, and their antioxidant activities.

## Materials and methods

### Plant material and growth conditions

For this study, we used 404 genotypes (accessions) of the potato CCC germplasm bank previously characterized at the molecular level using SNPs by Berdugo-Cely et al. (2017). The analyzed population held 320 tetraploid accessions (2n=4x=48) and 84 diploid accessions (2n=2x=24) genotypes. Of these materials, 335 are Colombian natives, 33 are foreign genotypes from Bolivia, Ecuador, the United States, Netherlands, and Peru, and 33 are of unknown geographic origin (**Supplementary Table S1**). In May of 2014, sixteen individuals per accession were grown in field conditions in Zipaquirá, Cundinamarca, Colombia (5° 03” 34.36”; N. 74° 03” 29.61 W). The location in Zipaquirá is at 2950 masl. presents clay loam textured soils with a pH of 5.5, relative humidity of 75%, and an average temperature of 15°C. During the harvest period, for each diploid (in September) and tetraploid (in November) type of potatoes, ten randomized tubers with weights between 60 and 80g with high physical and sanitary quality traits per accession were collected. These tubers were washed and sent for chemical analysis to the Laboratorio de Ciencia de los Alimentos of the Universidad Nacional de Colombia situated in Medellín (Antioquia) (https://direcciondelaboratorios.medellin.unal.edu.co/index.php/nuestros-laboratorios/facultad-de-ciencias/22).

### Phenotyping

#### Sample preparation

We cut one section of each tuber per accession collected (10 tubers); these sections were mixed and macerated. From this tissue, five grams (g) were mixed with 25mL of 80% methanol and shook at 500 rpm per 60 min. The mixtures were then centrifuged at 3000 rpm for 15 min. Subsequently, the suspensions were filtered and conserved at four (4) °C until further analysis (approximately between one and five days). Three suspensions per potato accession were independently prepared to measure all traits, constituting three technical replicates.

#### Total phenol content

The TPC was assayed using a modified protocol for the Folin-Ciocalteu method (Singleton and Rossi, 1965). Fifty microliters (μL) of filtered sample suspension, 425μL of dd H_2_O, and 125μL of Folin-Ciocalteu reagent were mixed. After five min of mixing, 400μL of 7.1% Sodium carbonate (Na_2_CO_3_) was added, and the mixtures were conserved for one hour in dark incubation at room temperature; after this time, the absorbance was measured at 760nm using gallic acid as a standard. The TPC was expressed in terms of milligrams of Gallic Acid Equivalent (GAE) per 100g of fresh weight (FW) (mg GAE/100g FW).

### Antioxidant activity

#### DPPH assay

A modified protocol for DPPH (*2.2-Diphenyl-1-picrylhydrazyl Radical Scavenging Activity*) assay was used to assess the AA (Brand-Williams et al., 1995). Nine hundred and ninety microliters of DPPH solution with 10μL of sample suspension were mixed and incubated for 30 min in the dark. After this time, the absorbance was measured at 517nm using Trolox (*6-hydroxy-2.5.7.8-tetramethylchroman-2-carboxylic acid*) reagent as a standard. The DPPH results (AA_DPPH) were expressed in terms of micromoles of Trolox Equivalent (TE) per 100g of FW (μmol TE/100g FW).

#### FRAP assay

The second assay to assess the AA was a modified protocol for FRAP (*Ferric Reducing Antioxidant Power*) assay (Benzie and Strain, 1996). Fifty microliters of sample suspensions were mixed with 50μL of 0.3μM acetate buffer (pH 3.4) and 900μL of FRAP solution (10 mM of TPTZ (*2.4.6-tripiridil-s-triazine*), 20 mM of Ferric chloride (FeCl_3_6H_2_O), 0.3 μM acetate buffer at pH 3.6). The mix was incubated for 30 min in the dark at room temperature. After this time, the absorbance was measured at 590nm using L-ascorbic acid as a standard. The FRAP results (AA_FRAP) were expressed as milligrams of Ascorbic Acid Equivalent (AAE) per 100g of FW (mg AAE/100g FW).

#### Ascorbic acid content

The AAC was evaluated through the modified method of Kelebek et al. (2009). Twenty microliters of the sample were filtered with a membrane filter of 0.45μm (Millipore) before being analyzed by High-Performance Liquid Chromatography (HPLC) in a liquid Shimadzu chromatograph (LC-20AD) equipped with an autoinjector SIL-20A/HT and a photodiode array (PDA) detector (SPD-M20A). A LiChrospherR 100 RP-18 column (Merck) (250 x 4 mm, particle size=5 LiChrospherR 100 RP-m) was used for HPLC separation, using a mobile phase of 0.1% of formic acid with flow rate of 0.8 mL/min at 35°C. The AAC concentrations were quantified compared to the L-ascorbic acid reagent as a standard. The AAC results were expressed as milligrams of Ascorbic Acid per 100g of FW (mg AA/100g FW).

#### Phenotypic statistical analysis

Means and standard deviations of the values obtained for TPC, AAC, and AA (AA_DPPH and AA_FRAP) were calculated. The Shapiro-Wilk test was used to check the normality of the data. One-way analysis of variance (ANOVA) and Tukey’s test (*p*<0.05) were conducted using the shapiro.test, aov, and TukeyHSD functions of *R-stats* package (R Stats, 2022) of R software (R, 2022) to 1) compare the means between and within populations for each variable and 2) compare the variations of TPC, AA, and AAC contents according to skin and flesh tuber colors (**Supplementary Table S1**). The tuber phenotype color traits were previously obtained (Berdugo-Cely et al., 2017) using the descriptors of the CIP (Centro Internacional de la Papa) to characterize native potatoes (Gómez, 2014). The following statistical analyses using the quantitative traits were independently run in diploid and tetraploid genotypes. Using the *R-stats* package (R Stats, 2022) a Pearson correlation (*p*<0.05) was used to check the relationship among variables. A Principal Component Analysis (PCA) was performed, and the two first components of the PCA were used to generate a multivariate clustering analysis using the Ward method and the Euclidean distance. These multivariate analyses were implemented in the *R-factoextra* package (Kassambara and Mundt, 2020).

### Genotyping

#### Genotype calling and filtering SNP data

The 404 potato accessions used in this study were previously genotyped in the study of Berdugo-Cely et al. (2017) using the Illumina SolCAP 8K SNP array. This chip array included 8303 SNP markers selected by EST (Expressed Sequence Tag) and transcriptomic data from five cultivars (Bintje, Kennebec, Premier Russet, Shepody, Snowden, and Atlantic) developed in the United States (Hamilton et al., 2011; Felcher et al., 2012). Here, the fluorescence intensities were extracted from Illumina GenomeStudio software (GenomeStudio Software, 2022), and the data was used to determine the genotype calling using *fitTetra* R-package (Voorrips et al., 2011) using with *SaveMarkersModels* function with the parameters of filtering reported by Vos et al. (2015). In the genotype calling, diploids were included allowing the corrected assigned of nulliplex (AAAA), duplex (AABB), and quadruplex (BBBB) clusters. Tetraploid genotypes were coded as: AAAA (0), AAAB (1), AABB (2), ABBB (3), BBBB (4), and diploid genotypes as: AA (0), AB (2), BB (4). Monomorphic markers, genotypes, and markers with missing data up to 20% and data with Minimum Allele Frequency (MAF) lower than 5% were filtered (**Supplementary Table S2**).

#### Population structure, genetic diversity, and linkage disequilibrium analysis

The genetic structure analyses based on the Bayesian clustering simulation in *STRUCTURE* software (Pritchard et al., 2000) were implemented on 1) the mixed population composed of diploid and tetraploid accessions and conducted separately on 2) diploid accessions, and 3) tetraploid accessions to assess the effect of various ploidy levels on the reconstruction of the population stratification. These analyses were simulated from one to ten subpopulations with five replicates for each dataset, assuming an admixture model and correlated allele frequencies with a burn-in of 100000 and 50000 Markov Chain Monte Carlo (MCMC) iterations after the burn-in. The number of subpopulations among the samples was determined using the Evanno method (Evanno et al., 2005) in *STRUCTURE Harvester* (Earl and vonHoldt, 2012). The PCA was conducted in the *adegenet* R-package (Jombart and Ahmed, 2011) using the identified genetic structure, which was confirmed by an analysis of molecular variance (AMOVA´s) and Phi values with 1000 permutations in the *poppr* R-package (Kamvar et al., 2014). The expected heterozygosity (He) was calculated to analyze the genetic diversity in each population using the *adegenet* package. We determined the linkage disequilibrium (LD) of the diploid, tetraploid, and mixed potato populations following the methodology reported by Vos et al. (2017). For the SNPs with a known physical position in the reference genome, all possible pairwise correlation coefficients (*r^2^*) were computed. The LD mean and decay per population were calculated using this information. The LD decay was determined by plotting the *r^2^* values against the physical distance and visualizing it using the *ggplot2* package in the R program (R, 2022).

#### Multivariate analysis and selection of promising diploid and tetraploid genotypes using phenotypic and genomic information

A PCA analysis that included the molecular and phenotypic information was implemented; in this case, the molecular traits correspond to the two first components of the PCA implemented with only molecular information and phenotypic information corresponding to the means values generated for each genotype in each feature (TPC, AA_DPPH, AA_FRAP and, AAC). A new cluster analysis was developed, and the genetic groups were described at the phenotypic level. Promising genotypes to TPC, AAC, and AA of the AGROSAVIA collection were identified and selected using an Index Selections (IS) calculated through the *selection index* R-package (Goyani, 2021), adjusting the weights of each character according to their heritability (*h^2^*) that was computed from phenotypic and genotypic data using the *heritability* R-package (Kruijer, 2019).

#### Genome‐wide association studies

The GWAS analyses were conducted using the mean values of TPC, AAC, and AA (AA_DPPH and AA_FRAP) obtained for each genotype. These analyses were implemented in the mixed population and independently in diploid and tetraploid populations because the CCC has a clear population genetic structure in function to the ploidy level (Berdugo-Cely et al., 2017). The complete dataset was analyzed under a tetraploid approach, while the diploid and tetraploid datasets were analyzed under their respective ploidy model approach. This study implemented the GWAS analyses through single-locus and multi-locus methods in the *GWASpoly* (Rosyara et al., 2016) and *mrMLM* (Zhang et al., 2020) R-packages, respectively. Kinship (K) matrices and population structure were included as random and fixed effects to remove associations related to the genetic structure.

A mixed linear model (MLM) implemented in *GWASpoly* was carried out for the single-locus method using the eight available models in this R-package (general, additive, diplo-general, diplo-additive, 1-dom-alt, 1-dom-ref, 2-dom-alt, and 2-dom-ref) (Rosyara et al., 2016). The *p*-values were adjusted with the False Discovery Rate (FDR) method (Benjamini and Hochberg, 1995) at *p*<0.001 to reduce the false-positive rate for the marker-trait association. On the other hand, in the multi-locus GWAS implemented in *mrMLM*, six models (mrMLM, FASTmrMLM, FASTmrEMMA, pLARmEB, pKWmEB, and ISIS EM-BLASSO) were evaluated (Zhang et al., 2020) using the default parameters. The threshold values to determine associated markers are usually established by corrections methods (e.g., Bonferroni), but those are sometimes too strict, missing some essential associations (Yousaf et al., 2021). Some studies (Phan et al., 2018; Riaz et al., 2018; Yousaf et al., 2021) have instead used a threshold of 3.0 or higher to determine the significant SNP-trait associations (STAs) or Quantitative Trait Nucleotide (QTNs). For this study, we established that STAs or QTNs with values of LOD (logarithm of the odds) scores higher than 3.5 were significantly associated. We reported LOD scores identified after evaluating all models for each STA (detected in a single-locus model) or QTN (detected in multi-locus models). The associated SNPs reported in this study were detected for a minimum of two models in each GWAS model. We constructed the Quantile–Quantile (q–q) and Manhattans plots for each evaluated model in R software. We graphed the plots using the model that detected the highest number of STAs in *GWASpoly*, while the graphs reported to *mrMLM* included the QTNs detected in all multi-locus models.

#### Candidate genes

The STAs and QTNs detected in common by single and multi-locus models (LOD score > 3.5) were searched against the potato genome of *Solanum tuberosum* group Phureja DM1-3 PGSC v4.03 in the Spud database - Potato Genomic Resource (PGSC Data Download; Xu et al., 2011; Sharma et al., 2013). Those genes were proposed as candidate genes associated with TPC, AA (AA_DPPH and AA_FRAP), and ACC.

## Results

### Total phenol content, ascorbic acid content and antioxidant activity in potato populations

**Figure 1:**
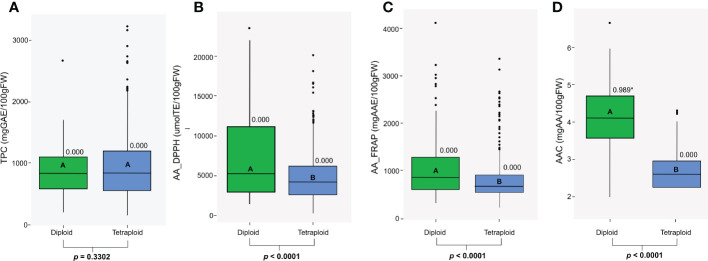
Distribution of **(A)** Total Phenol Content (TPC), **(B)** Antioxidant Activity measured by a DPPH assay (AA_DPPH), **(C)** Antioxidant Activity measured by a FRAP assay (AA_FRAP), and **(D)** Ascorbic Acid Content (AAC) in diploid and tetraploid potato populations. The *P-value* for the Shapiro-Wilk normality test is shown, and significant values (*p*>0.05) are specified with an asterisk. The letters and *p-values* in bold boxplots indicate a significant difference between populations based on the ANOVA and the Tukey test (*p*<0.05).

We studied antioxidant properties evaluating the CCC for TPC, AAC, and AA. Diploid and tetraploid genotypes in the CCC varied substantially in the TPC, AA, and AAC traits. These values did not follow a normal distribution (p<0.05) except for AAC in the diploid population (*p*=0.989) (**Figure 1**). Within diploid and tetraploid populations, all traits presented a wide range of phenotypic diversity with highly significant differences (*p*<0.0001) (**Figure 1**). Differences in TPC values were not significant between diploid and tetraploid populations (*p*=0.3302), but for some tetraploids, TPC was higher. Therefore, the range for tetraploids was wider (144.65 to 3218 mg GAE, median: 838.06) than in diploids (189.4 to 2584.4 mg GAE; median: 826.245) genotypes (**Figure 1A**). Values for AA using the DPPH assay ranged from 436.37 to 23677.38 μmol TE in diploid genotypes (median: 5287.61), and between 192.85 to 20243.40 μmol TE (median: 4248.82) in tetraploid genotypes (**Figure 1B**), while values for AA using the FRAP assay ranged from 271.26 to 4086.43 mg AAE (median: 831.81) in diploid genotypes and between 167.74 to 3333.90 mg AAE in tetraploid genotypes (median: 634.5) (**Figure 1C**). Diploid genotypes presented significantly higher values for AAC (*p*<0.0001) and AA measured by FRAP (*p*<0.0001) and DPPH assay (*p*<0.0001). Finally, AAC values ranged from 1.94 to 6.61 mg AA in diploid genotypes (median: 4.04) and between 1.65 to 4.26 mg AA in tetraploid genotypes (median: 2.54) (**Figure 1D**). Diploid and tetraploid differed in their antioxidant compounds concentration; diploid potatoes had significantly higher values for AAC and AA, while tetraploid genotypes had higher but non-significant values of TPC.

### Correlations among TPC, AAC, and AA in potato populations

**Figure 2:**
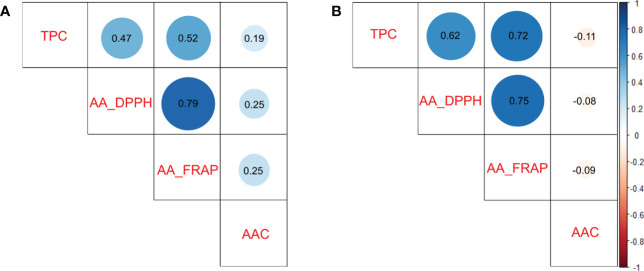
Pairwise Pearson correlation between Total Phenols Contents (TPC), Ascorbic Acid Content (AAC), and Antioxidant Activity (AA_DPPH and AA_FRAP) in potato populations. **(A)** Diploid population and **(B)** Tetraploid population.

To understand which compound could have more influence on antioxidant activity, we calculated pairwise Pearson correlations between traits. The TPC and AA traits presented intermedium and high significant positive correlations (*p*<0.0001) in diploid (*r^2^* >0.47 in TPC *vs* AA_DPPH) and tetraploid (*r^2^* >0.62 in TPC *vs* AA_DPPH) populations, respectively. AA measured by DPPH and FRAP assays presented high significant positive correlations (*p*<0.0001) in both populations (*r^2^* >0.75). In contrast, low or no significant correlations were detected between TPC and AAC (*r^2 =^* 0.19 in diploids and *r^2^* <-0.11 in tetraploids) and between AAC and AA (*r^2^* <0.25 in diploids and *r^2^* <-0.09 in tetraploids) in both populations (**Figures 2A, B**). Based on the results, TPC appeared to be essential in AA in potatoes.

### Phenotypic variation of TPC, AAC, and AA related to tuber color in potato populations

To evaluate if antioxidant content and properties differ depending on skin and flesh tuber color, we conducted an ANOVA and a Tukey Test, independently for tetraploids and diploids. TPC and AA levels presented significant differences (*p*<0.002) depending on the skin tuber color. In the tetraploid population, purple-blackish tubers had higher levels of TPC and AA (**Supplementary Figures S1A–C**), and diploid pink-red potatoes presented high and significant values of AA measured by FRAP assay (*p*<0.05) (**Supplementary Figure S1C**). In contrast, the AAC did not seem to depend on the skin tuber color in any population (**Figure S1D**). Moreover, the TPC, AA, and AAC in potatoes did not differ concerning the color of the flesh tuber in the analyzed populations (**Supplementary Figure S2**). From our results, in potatoes, TPC and AA change depending on skin tuber color, but AAC no.

### Phenotypic multivariate analysis in diploid and tetraploid populations

**Figure 3:**
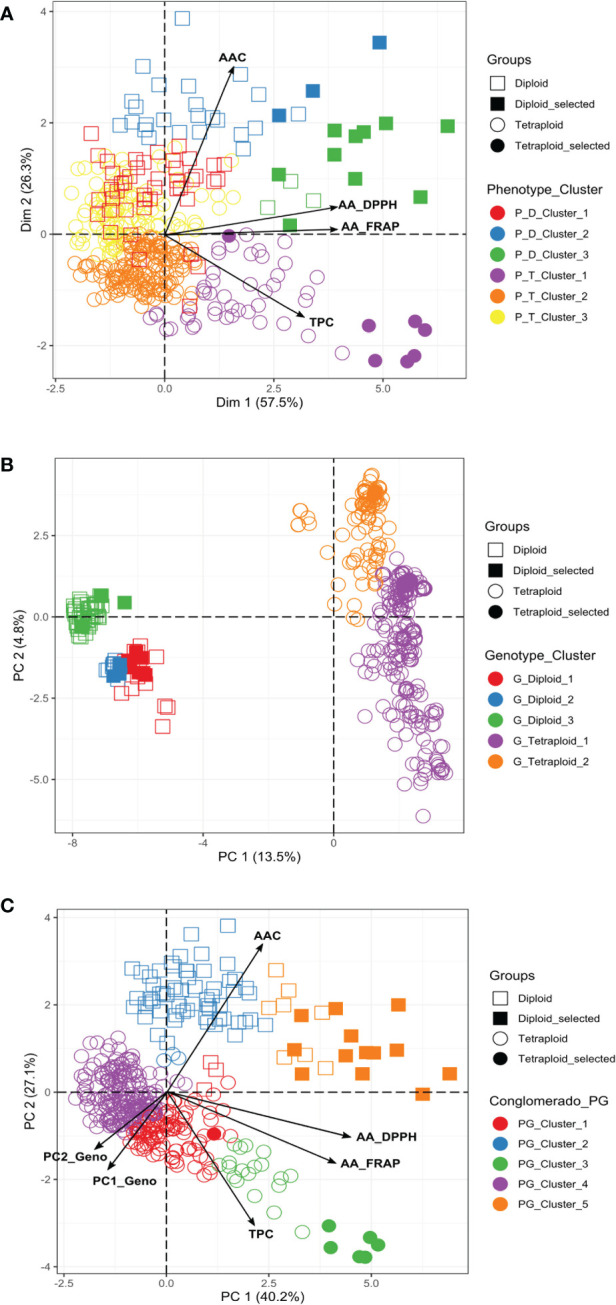
Principal Component Analysis (PCA) based on TPC, AA, and AAC phenotypic data and polymorphic molecular markers (4488 SNPs) for diploid and tetraploid accessions. **(A)** Phenotypic PCA **(B)** Genotypic PCA, and **(C)** PCA based on phenotypic and genotypic data. P, Population; D, Diploid; T, Tetraploid; G, Genotypic; PG, Phenotypic/Genotypic. The promising selected genotypes identified in this study were indicated in the figures. Additionally, the clusters showed in the figures were obtained from the phenotype, genotype, and phenotype/genotype multivariate analyses.

We conducted a PCA to understand which components of the phenotypic data explain most of the variation. The two first components of the PCA explained more than 80% (Dim1: 57.5% and Dim2: 26.3%) of the total variation for the diploid and tetraploid potato populations in the multivariate analyses (**Figure 3A**). AA and TPC were the most informative traits in the first component of the PCA, explaining between 57-61% and 48-56% of the variation, respectively. AAC was the most informative trait in the second component explaining between 91-99% of the variation. The phenotypic PCA and the cluster analysis confirmed that diploids had higher values of AAC and AA, and tetraploids tend to have higher values of TPC (**Figure 3A**). The phenotypic traits grouped the diploid and tetraploid samples in three clusters (**Supplementary Table S3**). In diploids, the genotypes with pink-red potatoes (n=13) with the highest values for TPC (mean=1352.92 ± 486.03 mg GAE), AA_DPPH (mean=16589.91 ± 4336.02 μmol TE), and AA_FRAP (mean=2432.5 ± 800.45 mg AAE) were clustered in the P_D_Cluster_3, while the genotypes with pink-red and purple blackish potatoes (27) with highest values for AAC (mean=4.92 ± 0.55mg AA) conformed the P_D_Cluster_2. In tetraploids, the genotypes with purple-blackish potatoes (52) with the highest TPC contents (mean=1817.94 ± 527,43 mg GAE), AA_DPPH (mean=9319.59 ± 4316.93 μmol TE) and AA_FRAP (mean=1463.31 ± 689.67 mg AAE) values were clustered in P_T_Cluster_1, while the genotypes with pink-red and purple-blackish potatoes (109) that present the highest AAC values (mean=3.09 mg AA ± 0.47) were found in P_T_Cluster_3 (**Supplementary Table S3**). We could recover clusters that differ in their TPC, AAC, and AA values.

### Genetic diversity, population structure, and linkage disequilibrium in potato populations

**Figure 4:**
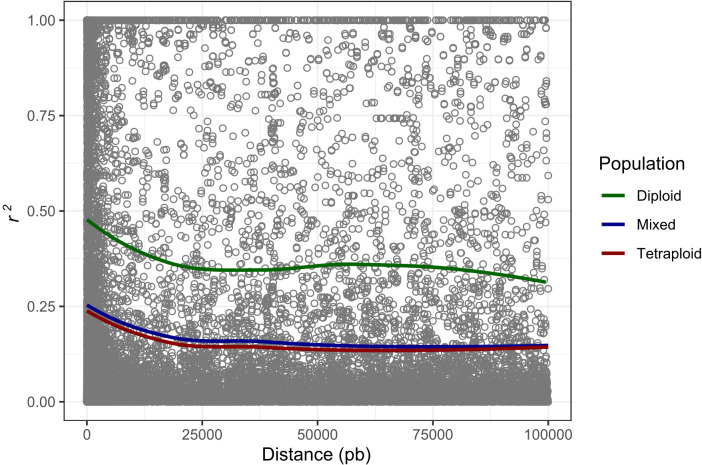
Genome-wide LD decay calculated to mixed, tetraploid, and diploid populations. The x-axis represents the physical distance in base pairs (pb) and the y-axis represents the average pairwise correlation coefficient (*r^2^*) of SNPs.

The potato germplasm’s genetic diversity and population structure analyses were analyzed using 4488, 2207, and 4487 polymorphic SNPs to assess the mixed, diploid, and tetraploid populations (**Table 1**). The mixed population was divided into two main subpopulations, the diploid and tetraploid subpopulations (**Figure 3B** and **Supplementary Figure S3A**). These subpopulations had a clear genetic differentiation (Phi=0.405) and a high genetic diversity (He=0.405). Diploid accessions presented medium genetic diversity levels (He=0.177) and were separated into three subpopulations (**Supplementary Figure S3B**) with a low genetic differentiation (Phi=0.038) (**Table 1**). However, these three diploid subpopulations differed in TPC, AAC, and AAC values. The subpopulation G_Diploid_2 regrouped genotypes with yellow-orange and pink-red potatoes with the highest levels of TPC (mean=1126.3 ± 394.52 mg GAE) and AA measured by DPPH (mean=10538.85 ± 5858.06 µmol TE) and FRAP (mean=1248.1 ± 534.33 mg AAE) assays. Moreover, the three subpopulations presented similar values of AAC with means between 3.97 to 4.25 mg AA (**Supplementary Table S4**). On the other hand, tetraploid genotypes were divided into two highly diversified subpopulations (He=0.422) (**Supplementary Figure S3C**) with low genetic structure (Phi=0.040) (**Table 1**). At the phenotype level, these two tetraploid subpopulations presented similar values for TPC, AAC, and AA (**Supplementary Table S4**). At the linkage disequilibrium level, all SNPs detected in mixed, diploid, and tetraploid populations were used to calculate the LD mean (*r^2^*) and LD decay. These values were similar in mixed (*r^2 =^* 0.192 with a LD decay=12500 base pairs - pb) and tetraploid (*r^2 =^* 0.179 with a LD decay=12000 pb) populations, while the LD was higher in the diploid population (*r^2 =^* 0.403 with a LD decay=11500 pb) (**Table 1** and **Figure 4**). The populations with high levels of genetic diversity (mixed and tetraploid) presented a minor level of LD. In contrast, the diploid population presenting low genetic diversity presented the highest LD levels. These results suggest that the low genetic diversity and high LD in the diploid population can affect the statistical power to identify SNP associated with phenotype traits in this population. The number of genetic variants necessary to conduct association studies in this subpopulation may be higher than the one used in this study.

**Table 1:** Genetic diversity, structure population and linkage disequilibrium statistics in potato populations.

Population	Number of samples	Number of polymorphic markers	Number of subpopulations	AMOVA		Genetic diversity (He)	Phi	LD mean (*r^2^*)
				Source of variation	Variation (%)			
**Mixed**	404	4488	K=2	Among	40.50	0.371	0.405*	0.192
				Within	59.49	
**Diploid**	84	2207	K=3	Among	3.81	0.177	0.038*	0.403
				Within	96.18	
**Tetraploid**	320	4487	K=2	Among	4.07	0.422	0.040*	0.179
				Within	95.92	

* Significative at 1000 permutations.

### Multivariate analysis and selection of promising genotypes in diploid and tetraploid populations using phenotypic and genomic information

The collection was also analyzed by combining the phenotypic and genomic data. The PCA and clustering analyses that included 67% of the genetic variability explained in the two first components of the PCA (PC1: 40.2% and PC2: 27.1%) suggested the division of these genotypes into five genetic groups, three subgroups for tetraploid and two subgroups for diploid genotypes (**Figure 3C**). The tetraploid genotypes with purple-blackish tubers (n=25) with the highest levels of TPC (mean=2062.41 ± 547.37 mg GAE), AA_DPPH (mean=12208.7 ± 2954.61 μmol TE), AA_FRAP (mean=1984.8 ± 622.53 mg AAE) and AAC (mean=2.46 mg ± 0.68 AA) were clustered in the PG_Cluster_3, while the diploids genotypes with pink-red tubers (n=21) with the highest levels of TPC (mean=1245.95 ± 426.82 mg GAE), AA_DPPH (mean=14967.1 ± 4687.79 μmol TE), AA_FRAP (mean=2208.63 ± 797.35 mg AAE) and AAC (mean=4.52 mg ± 0.68 AA) were grouped in the PG_Cluster_5 (**Supplementary Table S5**).

The selection index (SI) for each trait was calculated based on heritability values (*h^2^*: TPC=0.6956; AA_DPPH=0.6505; AA_FRAP=0.466 and AAC=0.6369). The tetraploid accessions, And_424, And_186, And_210, And_18, and And_209, had higher SI values (SI>1845) and concentrations for TPC, AA (DPPH and FRAP), and AAC traits with a concentration of phenolic compounds above 2651 mg GAE. We also selected the tetraploid accessions And_210 and And_290, and diploids Cha_49, Cha_46, and Phu_42 genotypes with SI>11886 and antioxidant activities above 18271 μmol TE measured by DPPH. Tetraploids And_186 and And_209 and diploids Cha_49, Cha_15, and Cha_16 genotypes were selected (SI>1453) for presenting antioxidant activities above 3113 mg AAE measured by FRAP. Diploid accessions Cha_2, Cha_16, Phu_7, Phu_75, and Phu_48 were selected (SI>7) based on their ascorbic acid contents above 5.24 mg AA. The tetraploid accessions And_186, And_209, and And_210 and the diploids Cha_49 and Cha_16 genotypes are particularly interesting for presenting high values for more than two traits (**Figure 3A** and **Supplementary Table S1**). This strategy allowed us to select 11 genotypes as promising. Additionally, a selection index calculated using the joint data of the four traits allowed the addition of nine genotypes to the list of promising materials (SI>11431). In total, we selected 20 genotypes as promising genotypes (**Supplementary Table S1**).

### SNP-trait associations using single-locus GWAS analyses

**Figure 5:**
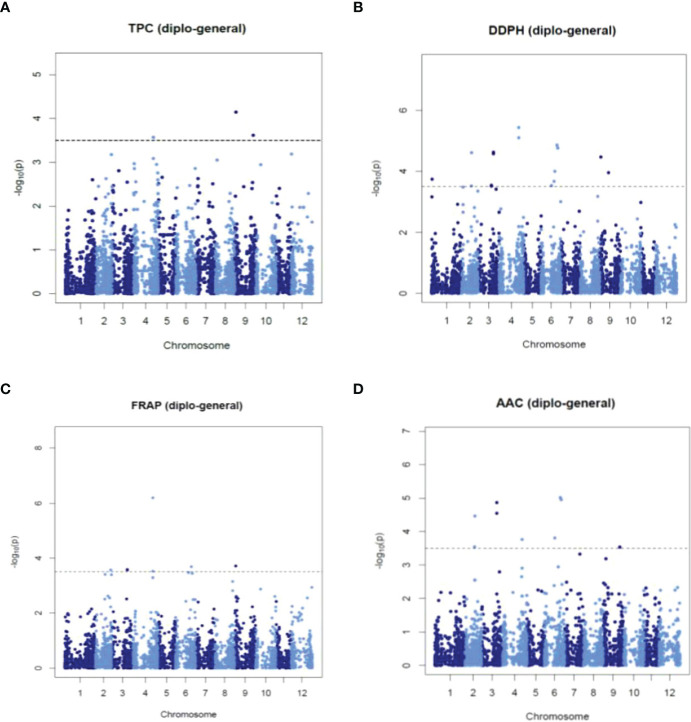
Manhattan plots showing significant STAs for TPC **(A)**, AA_DPPH **(B)**, AA_FRAP **(C)**, and AAC **(D)** obtained using single-locus GWAS methods in the mixed population.

A single-locus GWAS was performed in mixed, diploid, and tetraploid populations to identify STAs with TPC, AA_DPPH, AA_FRAP, and AAC. We considered an STA when at least two models with LOD score > 3.5 of *GWASpoly* identified it. The analysis revealed fifty-eight (58) STAs, 25 STAs in the mixed population, 16 STAs in diploid, and 17 STAs in tetraploid populations (**Supplementary Table S6**). Twelve STAs were pleiotropic because they were associated with two or three traits, while 17 were shown in two or three populations. Most STAs were revealed in only one population: 14 STAs in diploid, 17 in mixed, and 11 in tetraploid populations. Manhattans and q-q plots showed significant STAs with normal data distribution (**Figure 5** and **Supplementary Figures S4**-**S6**). In the mixed population, the analyses showed five STAs (in chromosomes 2, 4, and 9) with TPC with phenotype variation estimated (PVE-*r^2^*) of 0.2-4.4%, 17 STAs (in chromosomes 2-6 and 9) with AA_DPPH (0.1-7.1%), nine STAs (in chromosomes 2-4, 6 and 9) with AA_FRAP (0.1-7.8%) and nine STAs (in chromosomes 2-4, 6 and 9) with AAC (0.1-2.1%) (**Figure 5** and **Supplementary Table S6**). In the diploid population, the analysis revealed two STAs in chromosome 3 and 12 STAs in chromosomes 2, 6, and 9 associated with AA_DPPH (31.1%) and TPC (2.2-15%), respectively (**Supplementary Table S6** and **Supplementary Figure S5**). However, no associations with AA_FRAP and AAC were shown. In the tetraploid population, the analysis exhibited four STAs in chromosomes 2, 4, and 9 with TPC (1.3-6.6%), nine STAs in chromosomes 2, 4, 6, 8, 9, and 11 with AA_DPPH (1.7-15.9%), 12 STAs in chromosomes 1, 4, 6, 8, 9, and 11 with AA_FRAP (0.8-10.2%), and one STA in the chromosome 4 with AAC (5.4%) (**Supplementary Table S6** and **Supplementary Figure S6**).

### Quantitative-trait nucleotides using multi-locus GWAS analyses

**Figure 6:**
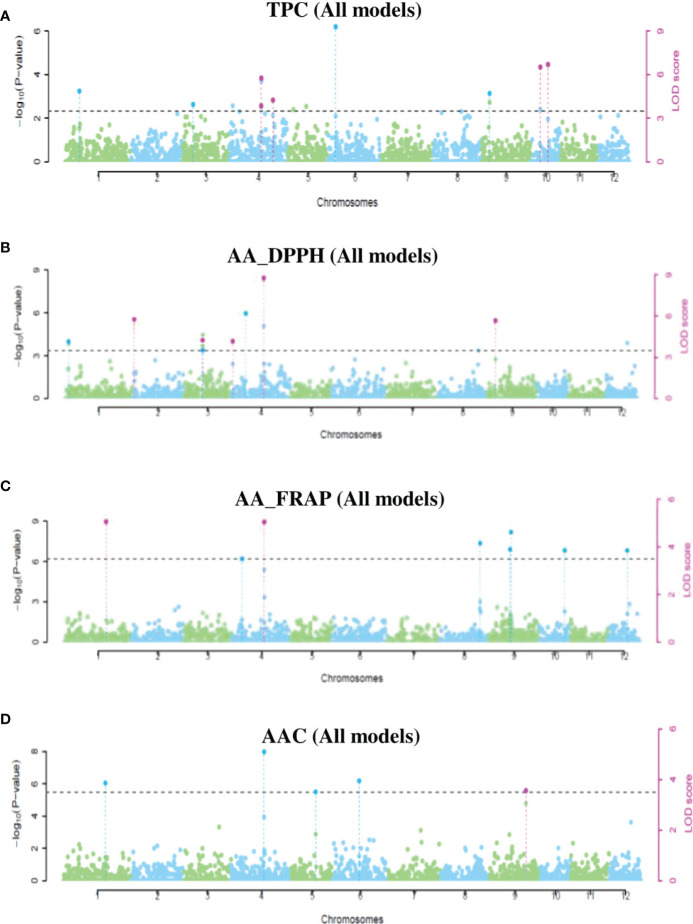
Manhattan plots showing significant QTNs for TPC **(A)**, AA_DPPH **(B)**, AA_FRAP **(C)**, and AAC **(D)** obtained using multi-locus GWAS methods in the mixed population.

Using the same three potato populations, we ran six models of multi-locus GWAS analyses. A total of 28 QTNs had significant associations with TPC, AA_DPPH, AA_FRAP, and AAC in at least two models implemented in *mrMLM*. This analysis revealed the highest number of QTNs in the tetraploid population (15 QTNs). In mixed and diploid populations, the analyses displayed 11 QTNs and two QTNs (**Supplementary Table S7**). Three QTNs were pleiotropic, and four QTNs were present in more than two populations. Most QTNs were revealed in only one population: two QTNs in diploid, nine in mixed, and 13 in tetraploid populations (**Supplementary Table S7**). We present the significant QTNs in Manhattan and q-q plots (**Figure 6** and **Supplementary Figures S7**-**S9**). In the mixed population, four QTNs in chromosomes 4 and 10 with a PVE*-r^2^* between 0.8-22.4% were associated with TPC, five QTNs in chromosomes 2, 3, 4, and 9 were associated with AA_DPPH (2.4-28.6%), two QTNs in the chromosomes 1 (5.1-32.5%) and 4 (10.4-11.0%) were associated with AA_FRAP. Finally, one QTN in chromosome 9 was associated with AAC (13.6-30.8%) (**Supplementary Table S7** and **Figure S6**). In the diploid population, only two QTNs had an association with TPC (25.76%) in chromosome 6 and AA in chromosome 6 with DPPH (63.8%) and FRAP assays (25.3%) (**Supplementary Table S7** and **Supplementary Figure S8**). In the tetraploid population, six QTNs associated with TPC were revealed in chromosomes 2, 4, 6, and 11, explaining between 4.6-26.8% of PVE. Three QTNs in chromosomes 1, 6, and 4 were associated with AA_DDPH (6.6-36.4%). Five QTNs were associated with AA_FRAP in chromosomes 1, 2, 4, and 12 (2.4-31.2%). Finally, only two QTNs in chromosomes one and 11 were associated with AAC (10.1-14.2%) (**Supplementary Table S7** and **Supplementary Figure S9**).

### Potential candidate genes revealed by single and multi-locus GWAS

We compared the single and multi-locus GWAS results to identify the more reliable genes associated with TPC, AA, and ACC. A total of eight STAs/QTNs were identified that flanked seven genes in chromosomes 1, 2, 3, 4, and 9 of the potato genome (**Table 2**). Four candidate genes were pleiotropic. The *PGSC0003DMG400024824* gene (Chr 4) of the CAZy family with a Glycosyltransferase function was detected by two STAs/QTNs (c1_12945 and c2_43998) associated with TPC (phenotype variation estimated PVE-*r^2^* 3.3-22.4%), AA_FRAP (2.1-36.4%), AA_DPPH (0.7-36.4%) and AAC (0.2-5.1%). The *PSC0003DMG400002675* gene (Chr 9) that has a fructose-bisphosphate aldolase function was associated with TPC (0.2-1.3%), AA_DPPH (4.8-24.7%) and AA_FRAP (4.2-4.9%). The *Soltu.DM.03G022550.1* gene (Chr 4) with a pectin methyl esterase function was associated with AA_DPPH (0.1-63.8%), AA_FRAP (22.7-25.3%) and AAC (0.1%) and the *PGSC0003DMG400001451* gen (Chr 2) with folate carrier protein were associated with TPC (13.9-22.6%) and AA_FRAP (7.8%). Additionally, three genes were associated with only one trait. The *Soltu.DM.01G033780.1* gen (Chr 1) with a cysteine synthase C1 function was associated with AA_FRAP (6.6-11%), the *Soltu.DM.03G015260.1* gene (Chr 3) with a BCL-2-associated athanogene function was associated with AA_DPPH (0.1-9.5%), and the *Soltu.DM.09G021110.1* gene (Chr 9) annotated as a basic helix-loop-helix (bHLH) DNA-binding superfamily protein was associated with AAC (0.6-30.8%) (**Table 2**).

**Table 2:** List of candidate genes obtained from STAs and QTNs detected in common in single and multi-locus GWAS methods with significant association with the content of phenolic compounds (TPC), ascorbic acid (AAC), and Antioxidant Activity (AA) in potatoes populations.

STA/QTN	SNP	Chrom	Position (bp)	Alleles	Number gene	Gene	Molecular Function	GWAS Model	Trait	LOD Scores	*R^2^* (%)
STA_QTN_1	c1_9566	1	72320482	A/G	Gen_1	Soltu.DM.01G033780.1	cysteine synthase C1	Single-locus: 3,4,6	AA_FRAP	3,5	6,6
								Multi-locus: 4,6	AA_FRAP	5,0579	10,4 - 11,0
STA_QTN_2	c2_15040	2	45649739	A/G	Gen_2	PGSC0003DMG400001451	folate carrier protein	Single-locus: 3,4,5	AA_FRAP	3,5	7,8
								Multi-locus: 4,5	TPC	4,8 - 7,7	13,9 - 22,6
STA_QTN_3	c2_48381	3	39198462	T/C	Gen_3	Soltu.DM.03G015260.1	BCL-2-associated athanogene	Single-locus: 3,6	AA_DPPH	3,5 - 3,7	0,1
								Multi-locus: 3,5	AA_DPPH	3,9 - 4,5	2,6 - 9,5
STA_QTN_4	c2_55279	3	45386107	A/C	Gen_4	Soltu.DM.03G022550.1	pectin methylesterase PCR fragment F	Single-locus: 2,4	AA_DPPH	3,6	31,1
								Single-locus: 1,3	AA_DPPH	3,9 - 4,5	0,1
								Single-locus: 1,3	AAC	3,5 - 4,5	0,1
								Multi-locus: 1,4,6	AA_DPPH	6,4205	49,7 - 63,8
								Multi-locus: 4,6	AA_FRAP	3,0091	22,7 - 25,3
STA_QTN_5	c1_12945	4	57899405	T/C	Gen_5	PGSC0003DMG400024824	Glycosyltransferase, CAZy family GT8	Single-locus: 1,3,4,5	AA_DPPH	4,6 - 6	0,7
								Single-locus: 1,3,4,5	AA_FRAP	5,4 - 6,8	2,1
								Single-locus: 3,4,5	AAC	3,5 - 4,4	0,2
								Single-locus: 1,5	AA_FRAP	3,5	2,4
								Single-locus: 1,3,4,5	AA_DPPH	3,5 - 4,7	4,1
								Single-locus: 1,3,4,5	AA_FRAP	4,8 - 4,7	5,1
								Single-locus: 4,5	AAC	3,9	5,1
								Multi-locus: 2, 3, 5, 6	TPC	3,5 - 4,8	3,3 - 10
								Multi-locus: 1,2,3,4,5,6	AA_DPPH	4,2 - 15,8	5,1 - 28,6
								Multi-locus: 1,2,3,4,5,6	AA_FRAP	3,6 - 15,4	5,1 - 32,5
								Multi-locus: 1,2,4,5,6	AA_DPPH	5,5 - 12,3	17,3 - 36,4
								Multi-locus: 1,2,3,4,5,6	AA_FRAP	3,8 - 11,3	5,2 - 31,2
STA_QTN_6	c2_43998	4	57899561	A/C				Single-locus: 4,6	AA_FRAP	3,7 - 4,0	0,2
								Single-locus: 2,3,4,6	TPC	3,5 - 4,2	4,4
								Multi-locus: 1,4	TPC	5,4 - 6,0	9,3 - 22,4
STA_QTN_7	c2_4030	9	3619115	T/C	Gen_6	PGSC0003DMG400002675	fructose-bisphosphate aldolase	Single-locus: 1,3,5	AA_DPPH	3,5 - 5,1	5,2
								Single-locus: 3,4,5	AA_FRAP	3,5 - 4,4	4,9
								Single-locus: 1,3,4,5	TPC	3,6 - 4,8	0,2
								Single-locus: 1,3,5	AA_DPPH	3,6 -5,2	4,8
								Single-locus: 1,3,4,5	AA_FRAP	3,6 - 5,4	4,2
								Single-locus: 3,5	TPC	3,7 - 4,4	1,3
								Multi-locus: 1,2,3	AA_DPPH	5,2 - 6,4	8,4 - 24,7
STA_QTN_8	c2_51155	9	49754441	C/T	Gen_7	Soltu.DM.09G021110.1	basic helix-loop-helix (bHLH) DNA-binding superfamily protein	Single-locus: 3,4	AAC	3,5 - 4,1	0,6
								Multi-locus: 1,2,4,5,6	AAC	3,5 - 4,7	13,6 - 30,8

**Single-locus models:** 1: General; 2: Additive; 3: Diplo-General; 4: Diplo-Additive; 5: 1-dom-alt; 6: 1-dom-ref; 7: 2-dom-alt; 8: 2-dom-ref. Multi-locus models: 1: mrMLM; 2: FASTmrMLM; 3: FASTmrEMMA; 4: pLARmEB; 5: pKWmEB; 6: ISIS EM-BLASSO.

## Discussion

The benefits in human health of consuming fruits and vegetables with high antioxidant properties are well known. The antioxidant compounds can protect against oxidative damage by scavenging ROS and reducing oxidation reactions. The phenolic compounds and ascorbic acid have antioxidant and anti-inflammatory properties, inhibiting, preventing, and treating various human diseases, including cardiovascular disease, neurodegenerative disorders, obesity, and hypertension (Arruda et al., 2020; Rahman et al., 2022). The antioxidant components extracted from potato tubers have prevented stomach, prostate, colon, and liver cancer and diseases such as type 2 diabetes (Burgos et al., 2019; Hellmann et al., 2021). In this study, we evaluated the total phenolic compounds (TPC) and ascorbic acid (AAC) contents and their antioxidant activities (AA) in a diverse panel of diploid and tetraploid potatoes genotypes to select promising genotypes to use in future breeding programs and to identify STAs and QTNs flanked in genes with a possible genetic association with the synthesis of TPC, and AAC, and their AA in potatoes.

### Diploid and tetraploid potato genotypes had differential values of TPC, AAC, and AA

In this study, we analyzed the influence of ploidy levels on the production of antioxidant compounds in potatoes. The analyzed accessions from AGROSAVIA’s collection showed a high variation in TPC, AAC, and AA values. This variation was associated with potatoes’ ploidy level and tuber colors; this collection conserves diploid and tetraploid native potatoes with a wide tuber phenotypic diversity of forms and colors (Berdugo-Cely et al., 2017). Here, the results showed that purple-blackish tetraploid and pink-red diploid potatoes had elevated levels of TPC and AAC with good antioxidant activity. In agreement with our results, previous studies reported a correlation between high levels of phenolic compounds, ascorbic acid, and antioxidant activity in potatoes and genotypes with skin and flesh dark colors tubers (Hu et al., 2012; Perla et al., 2012; Albishi et al., 2013). Skin and flesh potato colors can be considered phenotypic traits to identify genotype accessions with high nutritional values.

The ploidy level can affect TPC, AAC, and AA values. Here, diploid potatoes had a higher concentration of ascorbic acid and antioxidant activity than tetraploid genotypes. Tetraploid genotypes tended to have higher concentrations of phenolic contents, but this difference was not significant. Potato diploid genotypes can present up to 13 times more carotenoid content than tetraploid genotypes, suggesting that tetraploid potatoes have lower nutritional value than diploid genotypes (Lu et al., 2001). Similar results in native South American potato cultivars were reported by Brown (2008). Some reports indicate that polyploid genotypes of *Glycine max*, *Solidago canadensis*, and *Fagopyrum esculentum* generally produce a more diverse and higher secondary metabolite concentration than diploids, explained by the presence of multiple gene copies generated by chromosome and gene duplication (Zagoskina et al., 2018; Gaynor et al., 2020; Yang et al., 2021). Environmental and genotypic effects can affect the concentrations of secondary metabolites in plants (Yuan et al., 2013), and they could be species-specific. For instance, diploid genotypes of *Anchusa officinalis* and *Camellia sinensis* presented higher concentrations of secondary metabolites than polyploid genotypes (Zagoskina et al., 2018). However, other plant species did not show differences in secondary metabolite concentration associated with ploidy levels (Gaynor et al., 2020). Our results show the importance of including diploid genotypes in a potato breeding program to improve antioxidant traits for the generation of biofortified cultivars with high nutritional values.

Phenolic compounds, reducing agents with redox properties, are one of the main responsible for the antioxidant activity in plants (Roman et al., 2013). In our study, we found that TPC and AA are correlated in agreement with earlier potato studies (Hu et al., 2012; Pinhero et al., 2016; Kim et al., 2019). TPC is one of the main contributors to antioxidant activity. This result would support the use of TPC as an indicator to assess the AA in fruits and vegetables (Hesam et al., 2012). In contrast, TPC and AAC were not correlated in tetraploids and had a low correlation in diploids. Although ascorbic acid is a well-known antioxidant, we found a weak correlation between AAC and AA in tetraploids. These results coincide with studies in pineapple, papaya, plum, and tamarind, in which no correlations between AAC and AA were found (Almeida et al., 2011). In a study analyzing juices from different fruit species, the authors found that the antioxidant capacity needs the synergetic action of ascorbic acid and polyphenols; the AAC depended more on the polyphenol’s concentration than on ascorbic acid content (Nowak et al., 2018). In agreement with Nowak´s study, our results suggest that the antioxidant activity in potatoes is more governed by the phenolic compounds than by the ascorbic acid; results in berries and flaxseed were similar (Silva et al., 2013; Manganaris et al., 2014).

The selection indexes (SI) allowed the choice of 20 promising genotypes from AGROSAVIA’s collection (13 diploids and seven tetraploids) with mainly dark skin tuber color. Some of the selected materials can be attractive for a breeding program (And_186, And_209, And_210, Cha_49, and Cha_16) because they presented good levels of both TPC and AA. The heritability values varied between medium and high (*h^2 =^* 0.466-0.695), in agreement with previous reports (*h^2 =^* 0.413-0.657) (Tierno and Ruiz de Galarreta, 2018). The *h^2^* values support using selected materials as elite parentals to initiate crossing processes in a breeding program for TPC, AA, and AAC (Tierno and Ruiz de Galarreta, 2018). In this study, we obtained an absolute value of the global concentration of phenolic compounds. In a next step, we are interested to identify the individual compounds and will analyze the concentration of each specific phenolic compound. A complete assessment of the nutritional value of this germplasm should include the analysis of other antioxidant components such as chlorogenic, vanillin, p-coumaric acid, and caffeic acid, among others.

### Single and multi-locus GWAS revealed STAs and QTNs associated with TPC, AAC, and AA

In the present work, GWAS could dissect the genetic basis of the natural variation of phenolic compounds, ascorbic acid content, and antioxidant activity in potato tubers. So far, few studies have evaluated the genetic control of secondary metabolites in potatoes (Parra-Galindo et al., 2019; Parra-Galindo et al., 2021), and few reports studied the genetics of quality traits such as fry color and starch content (Schönhals et al., 2017; Byrne et al., 2020; Naeem et al., 2021). Then, until this moment, this could be the first GWAS that identified STAs and QTNs that flanked genes with a possible genetic association with TPC, AA, and AAC in potatoes using diploid and tetraploid genotypes. In other cultivated plant species, GWAS revealed genes associated with TPC in rice (Xu et al., 2016), apple (McClure et al., 2019), sorghum (Habyarimana et al., 2019; Kimani et al., 2020), barley (Han et al., 2018), and tomatoes (Ruggieri et al., 2014) and associated with AAC in tomatoes (Ruggieri et al., 2014; Sauvage et al., 2014; Ye et al., 2019).

Here, we implemented single and multi-locus GWAS analyses of the complete dataset that included diploid and tetraploid potatoes and also ran the analyses separating the potatoes by their ploidy level. The CCC has a strong population structure based on the ploidy level; this collection is structured into diploid and tetraploid potatoes (Berdugo-Cely et al., 2017). Ploidy levels have been reported as one of the main factors to explain the population structure in other potato collections (Stich et al., 2013; Hardigan et al., 2015; Berdugo-Cely et al., 2021). Identifying the genetic structure of panels used in association mapping analyses is essential to eliminate false marker-traits associations influenced by genetic relationships and population structure tendencies. These population characteristics must be included in the GWAS models to correct and exclude these false associations (Flint-Garcia et al., 2005; Zhu et al., 2008). To implement GWAS analysis, it is also necessary to identify the LD degree among SNPs in the population because these statistics would establish if the population is suitable for association mapping studies and the degree of the mapping resolution (Flint-Garcia et al., 2005; Yousaf et al., 2021). The LD mean value and the genetic diversity (Ho) of diploids contrasted with the values of the mixed and tetraploid populations. The same pattern of high LD and low Ho in diploid potatoes and low LD and high Ho in tetraploids was previously reported (Berdugo-Cely et al., 2017; Berdugo-Cely et al., 2021). As reported in other studies (Gebhardt et al., 2004; Simko et al., 2006), the LD decayed slowly in all populations, and it is explained by the clonal propagation of potatoes that limits the number of meiotic generations and, in consequence, the recombination events (Gebhardt et al., 2004; Simko et al., 2006). The LD pattern in potatoes makes possible the use of a modest number of SNPs, as included in the 8K potato SNP array, to find market-trait associations (Gebhardt et al., 2004; Simko et al., 2006). In this study, we confirmed that the number of SNPs was enough to find STAs and QTNs for the analyzed traits. However, the number of STAs and QTNs in the diploid population was minimal.

In the single and multi-locus GWAS analyses, the number of significant associations found for TPC, AAC, and AA (AA_DPPH and AA_FRAP) traits depended on the dataset/populations (mixed, diploid, and tetraploid) and the GWAS models (single and multi-locus) used. At the level of the dataset, the number of detected STAs and QTNs was higher in the mixed (in single locus GWAS) and tetraploid (in multi-locus GWAS) datasets than in the diploid dataset (in both GWAS models: single and multi-locus), probably due to the higher sample number in the mixed and tetraploid populations. The size population affects the obtention of reproducible and robust results in GWAS analyses (Uffelmann et al., 2021); for this, it would be necessary to increase the number of individuals in the diploid population to ensure that the number of genetic associations detected was not affected by this difference in population size. In sugarcane, a species with a complex population structure based on ploidy levels, GWAS identified the highest number of genetic associations in the complete dataset and the largest subpopulations. Associations detected in the subpopulations were not detected in the complete dataset, probably because of the population size and structure population (Yang et al., 2020). Similar results were found in our study; the highest number of the STAs and QTNs detected in the diploid and tetraploid populations were not detected in the complete dataset. This result suggests that GWAS cannot detect the overall genetic associations even with a correction by population structure in a mixed population. Then, GWAS in potatoes should be conducted using complete datasets and data separated by ploidy level to identify the highest number of genetic associations.

The single-locus GWAS detected more genetic associations than the multi-locus model. In contrast, the LOD scores and the phenotype variation estimated (PVE-*r^2^*) were higher in multi-locus than in the single-locus GWAS. The conventional single-locus GWAS, such as the general linear model (GLM) and mixed linear model (MLM), is considered less robust because it neglects the overall effects of multiple loci and needs corrections for critical values. QTLs with minor effects are sometimes not detected, and the effect estimations (*r^2^*) are less accurate with single-locus models. GWAS multi-locus methods would have lower false-positive error and higher statistical power in detecting a higher number of genetic associations (Wang et al., 2016; Cui et al., 2018; Hu et al., 2018; Zhong et al., 2021), as reported in studies in rice and cotton (Li et al., 2018; Zhong et al., 2021). Our results differ because we used the *GWASpoly* package, which implements models with different types of polyploid gene action, including additive, simplex dominant, and duplex dominant useful in the analysis of an autopolyploid species such as potato (Rosyara et al., 2016). We also used an FDR correction instead of a Bonferroni correction because Bonferroni is a very stringent correction method that identifies a minor number of genetic associations (Merrick et al., 2022). Another difference is that only QTNs detected by at least two models in the multi-locus GWAS were kept. Variations in GWAS analyses used in this study increase the probability of detecting a significant number of genetic associations with single-locus models. The single and multi-locus GWAS detected eight QTNs/STAs in common that flanked seven genes statistically associated with TPC, AAC, and AA. Implementing single and multi-locus GWAS methods allows to improve the power and robustness of association analyses (Li et al., 2018).

The phenolic compounds and the ascorbic acids are antioxidants and co-factors of many processes in plants. Genes implicated in their synthesis involve several biotic and abiotic stress responses in plants (Martínez-Lüscher et al., 2014) in congruence with our results, where four pleiotropic genes were associated with TPC and AAC with antioxidant activity responses. In the present study, the gene *PGSC0003DMG400024824* (Chr4) with a *Glycosyltransferase* function was associated with the three analyzed traits, TPC, AAC, and AA (AA_DPPH and AA_FRAP). This gene belongs to the *UDP-glycosyltransferase (UGTs) superfamily* that codifies proteins involved in the biosynthesis of phenolic compounds such as anthocyanins and flavonoids (Bowles et al., 2005; Dong et al., 2020). In rice, glycosyltransferases were associated with heat, drought, and salt stress responses (Obaid et al., 2016; Li et al., 2020; Liu et al., 2021). The *PGSC0003DMG400002675* (Chr 9) gene with a *fructose-1, 6-bisphosphate aldolase (FBA)* function was associated with TPC and AA (AA_DPPH and AA_FRAP). Recently, a possible association of the FBA with the phenylalanine biosynthesis of a phenolic compound in *Cannabis sativa* was reported (González Camargo et al., 2022). Enzymes with these functions also are mainly reported in wheat as a response to salt, drought, heat, and low-temperature stresses (Lv et al., 2017). The *Soltu.DM.03G022550.1* (Chr3) gene with a *pectin methylesterase (PMEs)* function was associated with AAC and AA (AA_DPPH and AA_FRAP). This enzyme has been associated with the production of ascorbic acid through the D-galacturonate pathway in tomato and tobacco (Rigano et al., 2018) and is involved in pectin remodeling, disassembly of the cell wall, and heat stress responses in Arabidopsis (Huang et al., 2017; Wu et al., 2018). The *PGSC0003DMG400001451* gene (Chr 2) with *folate carrier proteins* function was associated with TPC and AA_FRAP; these proteins are involved in the downregulation of folate biosynthesis as a response to cold in rice (Neilson et al., 2011; Gorelova et al., 2017).

Another three genes detected in common among single and multi-locus GWAS were associated with only one trait. The *Soltu.DM.09G021110.1* (Chr 9) gene that transcribed for *basic helix-loop-helix (bHLH) transcription factor (TF)* was associated with AAC. The bHLH TF acts as a co-regulator in the biosynthesis of phenolic compounds such as anthocyanins (Liu et al., 2016). But, in maize and tomato, bHLH TF regulates the expression of genes involved in ascorbic acid biosynthesis through the GDP-mannose/L-galactose pathway (Ye et al., 2019; Yu et al., 2021) and causes the accumulation of ascorbic acid under salt stress in maize (Yu et al., 2021). The bHLH TF has also been associated with water, salt, and drought stress responses in tobacco, maize, and peanut, respectively (Zhao et al., 2020; Li et al., 2021; Yu et al., 2021). The *Soltu.DM.01G033780.1* (Chr 1) gene with a *cysteine synthase C1 (CSase)* function was associated with AA_FRAP. This enzyme responds to stress caused by high-salt conditions, heavy metals, and pathogen responses in alfalfa and brassica (Xie et al., 2013; Yuan et al., 2022). Finally, the *Soltu.DM.03G015260.1* (Chr 3) gene with *Bcl-2-associated athanogene (BAG) family protein* functions are involved in heat stress responses in rice (Rashid et al., 2011).

Many detected STAs/QTNs presented minor effects, suggesting that multiple genes probably control the phenotypic traits. It is recognized that phenolic compounds and ascorbic acid are quantitative traits (Habyarimana et al., 2019; Kimani et al., 2020). However, some associations explained until 63.8% of the phenotypic variation of the evaluated traits. Similarly, in blueberries, genetic factors (>40%) controlling secondary metabolite concentrations (e.g., anthocyanins, flavanols, and phenolic acids) were reported (Mengist et al., 2020). The analyzed phenotypic traits had a high heritability enabling the implementation of MAS for these attributes in potatoes that could start using the four identified pleiotropic genetic associations. According to the genotype scoring data, these markers would allow the identification of potato individuals with elevated levels of these compounds and good antioxidant activity. It is then required to design and validate these molecular markers *via* fine mapping and expression analyses to determine their efficiency in selecting promising genotypes and their role in the genetic control of the traits analyzed here. In plant breeding, validated molecular markers are used for initial screening or selecting individuals from crosses between elite parents. Marker-assisted selection can be performed using easy and fast strategies based on PCR, such as KASP (Kompetitive Allele-Specific PCR) markers (Chen et al., 2021; Fu et al., 2021). In the future, it is necessary to select new associated genomic regions using trait evaluation data conducted in multiple locations for several years, as realized in wheat (Vikas et al., 2022) and peach (da Silva Linge et al., 2021). It is also necessary to explore other strategies, such as Genomic Selection (GS), to improve these features in potatoes.

## Conclusions

Genotypic and phenotypic results revealed that the potato germplasm conserved in AGROSAVIA holds valuable and promising genotypes with high levels of TPC and AAC with good antioxidant properties, especially the diploid genotypes. We identified higher values for the studied traits than those found in other studies, probably as an effect of the reduced number of potato accessions or breeding lines evaluated in those studies (Hesam et al., 2012; Tierno et al., 2015; Pinhero et al., 2016; Silva-Beltrán et al., 2017; Kim et al., 2019; Soare et al., 2020). We were able to select 20 genotypes as candidate materials for breeding programs looking to improve these traits in potatoes. From literature reports, we confirmed the nutritional importance of potatoes over other plant species, such as pineapple, apples, carrots, onions, tamarind, and tomatoes, as a source of TPC and AAC (Leja et al., 2013; Kschonsek et al., 2018; Sagar et al., 2020). However, the genotype-environment interaction affects the production of these compounds, making sometimes invalid the comparison between studies conducted in different countries and environments (Reddivari et al., 2007; André et al., 2009). Therefore, we recommend that future research projects evaluate AGROSAVIA’s collection in multiple producing regions of Colombia for several years to establish if TPC, AAC, and AA are affected by the environment and other variables. For instance, in blueberries, the secondary metabolite concentrations, despite having high heritability (>40%), were affected by the environment (Mengist et al., 2020). Evaluating morpho-agronomic and biochemical compounds in a high number of potato genotypes in the field in multiple locations for several years could be very expensive. Consequently, we recommend evaluating the CCC Core collection that includes 10% (128 genotypes) of the complete genetic diversity of the CCC (Manrique-Carpintero et al., 2022. Submitted). In this study, we also found that STAs and QTNs associated with TPC, AAC, and AA differed between mixed, diploid, and tetraploid populations and that analyses are affected by the population size and structure and the GWAS model used. GWAS in potatoes should be done in mixed ploidy populations and in populations separated by ploidy, to identify a major number of genetic associations. GWAS allowed the identification of seven candidate genes associated with phenolic compounds, ascorbic acid content, and antioxidant properties. Many of these associations were mapped in genes with molecular functions involved in abiotic stress responses. However, further functional validation is needed to confirm their biological role. In a breeding program, these associated genes would allow the implementation of MAS of potato materials with good antioxidant properties and tolerance to abiotic stresses (Burgos et al., 2019; Arruda et al., 2020; Hellmann et al., 2021; Rahman et al., 2022).

## Data availability statement

The SNP data used in this manuscript were provided in the supplementary data (**Supplementary Material Table S2**). The data was obtained from the potato SNP array. Further queries should be directed to the corresponding author.

## Author contributions

JB-C carried out phenotype and genotype statistical analyses, made graphics, and wrote the manuscript. MC-L achieved the funding for the biochemical process and contributed to revising the manuscript. RY contributed to interpreting data, editing the graphics, and writing and revising the manuscript. All authors contributed to the article and approved the submitted version.
