# Supplementary figures and images for: Phenotypic and molecular analyses in diploid and tetraploid genotypes of Solanum tuberosum L. reveal promising genotypes and candidate genes associated with phenolic compounds, ascorbic acid contents, and antioxidant activity
Source: Front Plant Sci. 2023 Jan 18;13:1007104. doi: 10.3389/fpls.2022.1007104 (PMC9889998; doi:10.3389/fpls.2022.1007104)

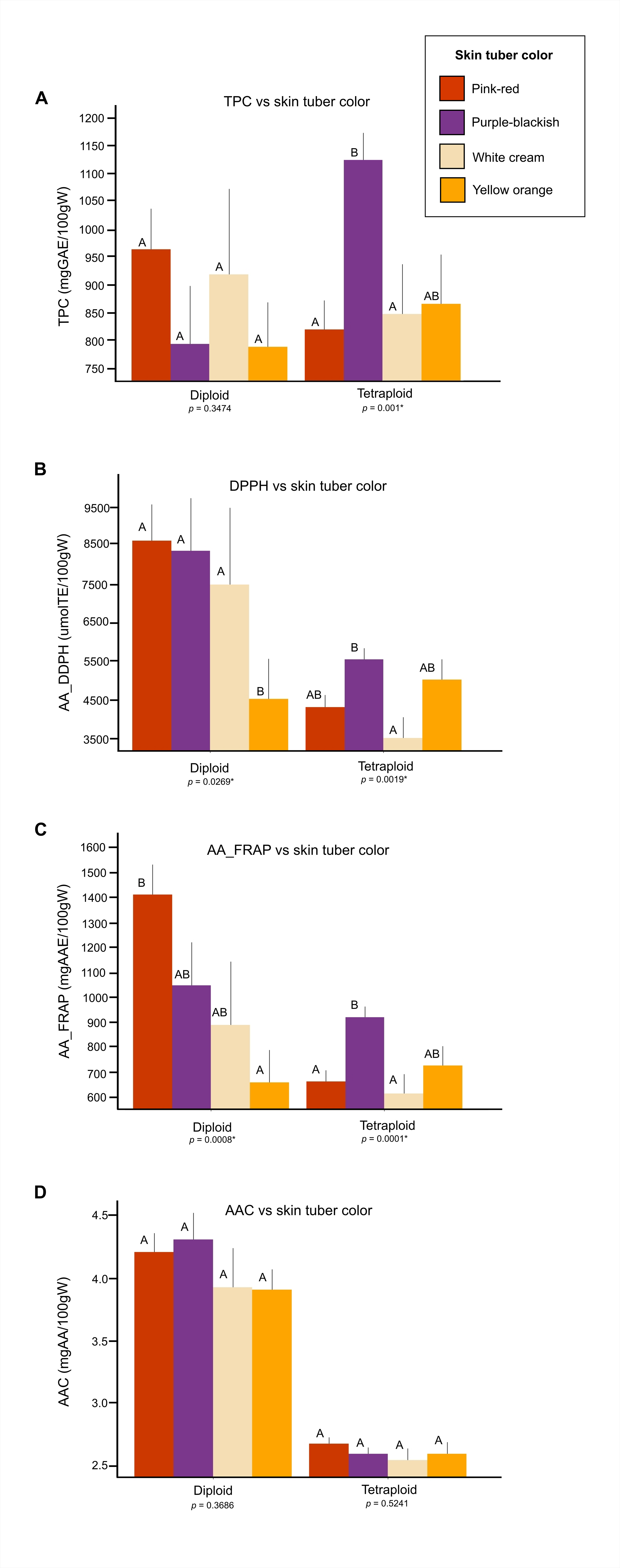

Supplement: Supplementary file 1 [file Image_1.tif]

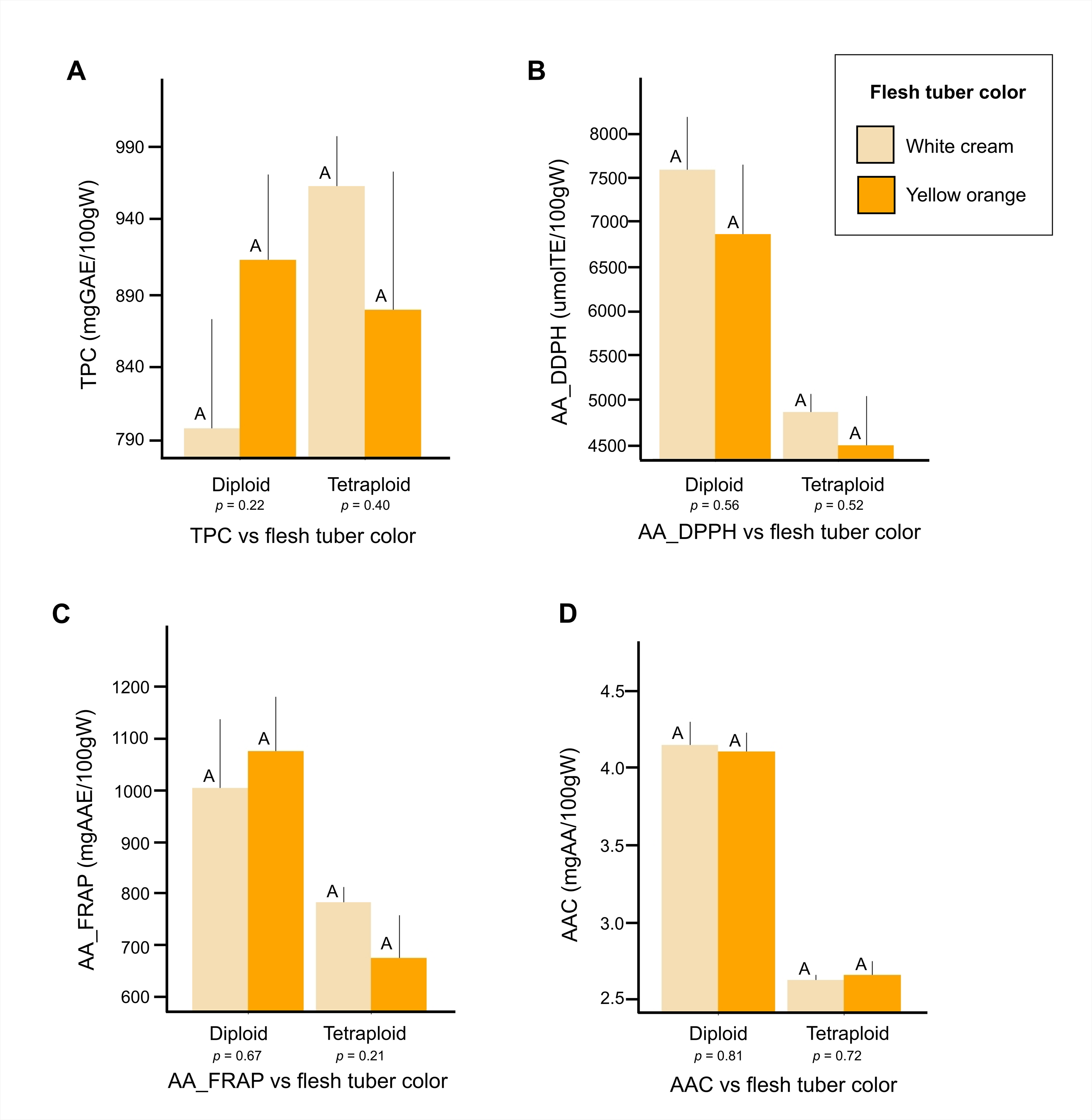

Supplement: Supplementary file 2 [file Image_2.tif]

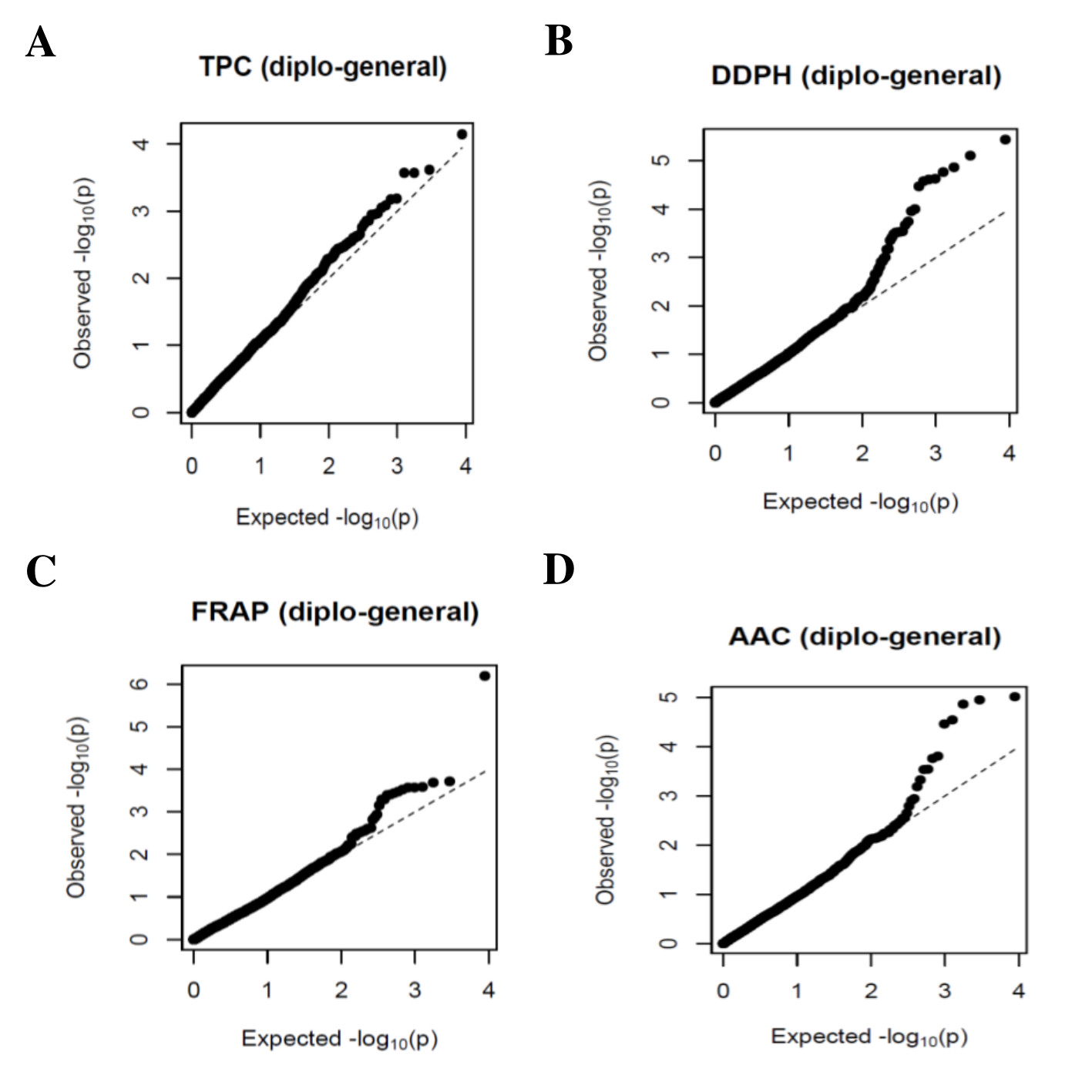

Supplement: Supplementary file 4 [file Image_4.tif]

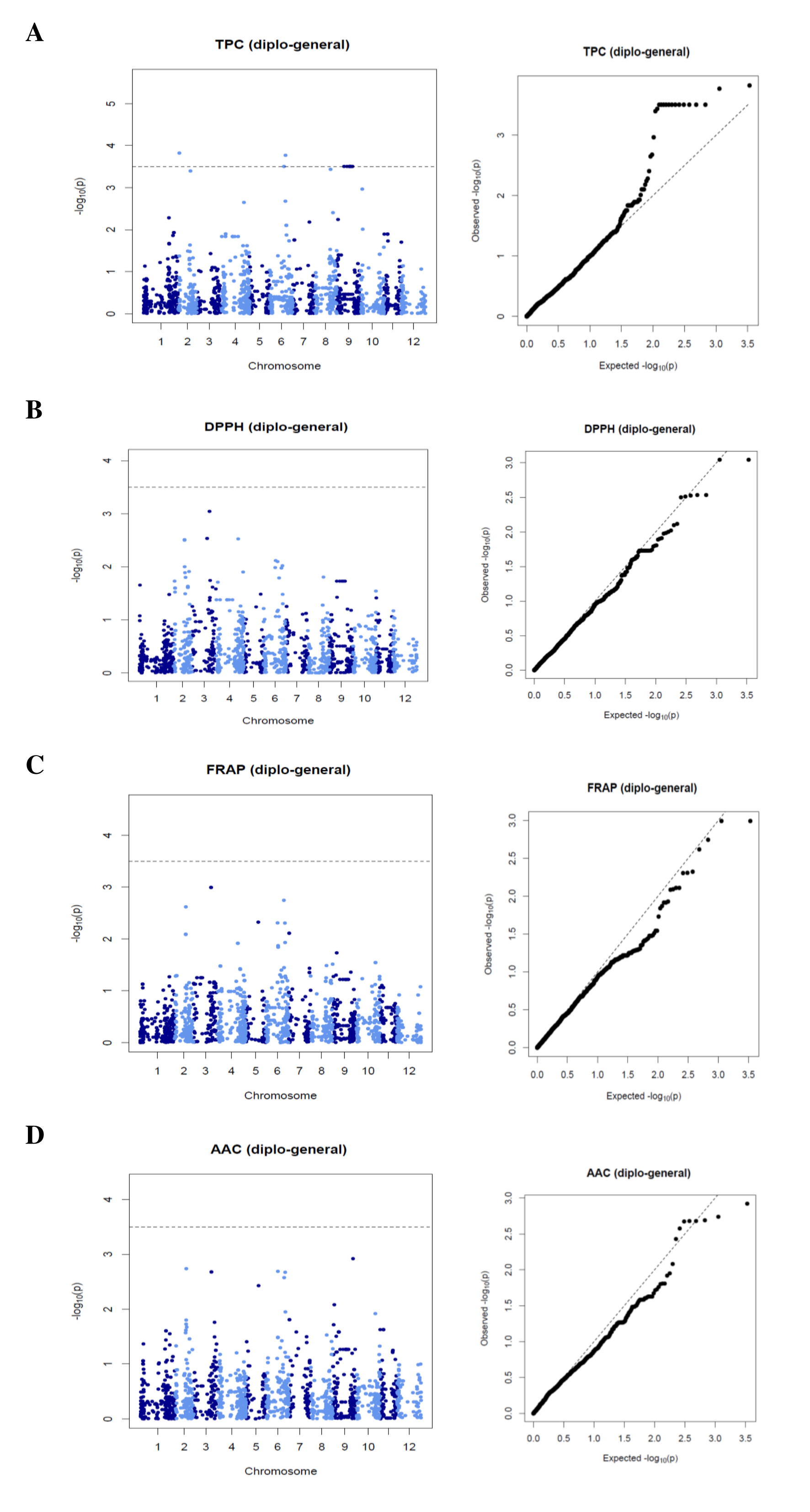

Supplement: Supplementary file 5 [file Image_5.tif]

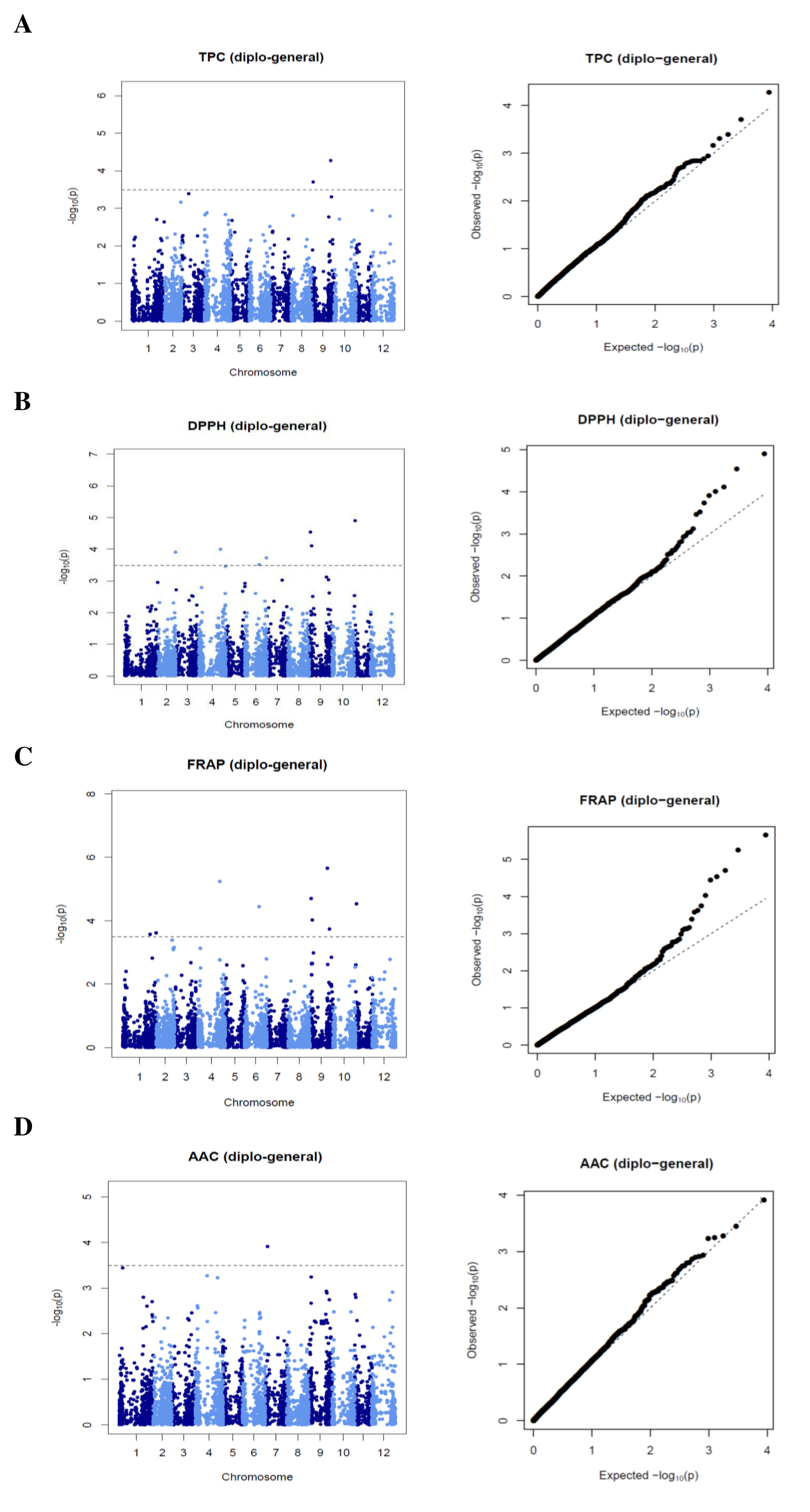

Supplement: Supplementary file 6 [file Image_6.tif]

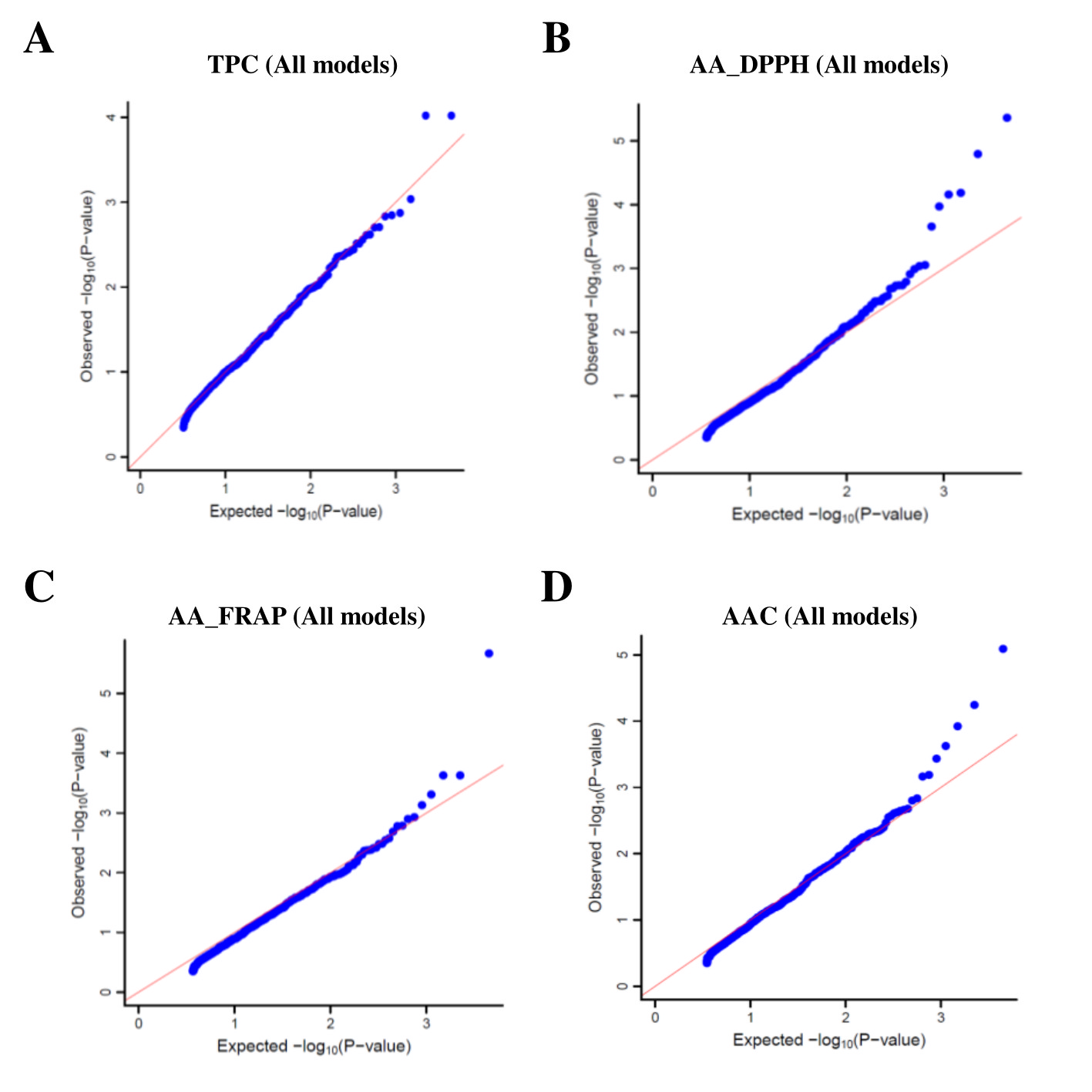

Supplement: Supplementary file 7 [file Image_7.tif]
